# Cellular Scale Anisotropic Topography Guides Schwann Cell Motility

**DOI:** 10.1371/journal.pone.0024316

**Published:** 2011-09-20

**Authors:** Jennifer A. Mitchel, Diane Hoffman-Kim

**Affiliations:** Department of Molecular Pharmacology, Physiology, and Biotechnology, Center for Biomedical Engineering, Brown University, Providence, Rhode Island, United States of America; Dalhousie University, Canada

## Abstract

Directed migration of Schwann cells (SC) is critical for development and repair of the peripheral nervous system. Understanding aspects of motility specific to SC, along with SC response to engineered biomaterials, may inform strategies to enhance nerve regeneration. Rat SC were cultured on laminin-coated microgrooved poly(dimethyl siloxane) platforms that were flat or presented repeating cellular scale anisotropic topographical cues, 30 or 60 µm in width, and observed with timelapse microscopy. SC motion was directed parallel to the long axis of the topography on both the groove floor and the plateau, with accompanying differences in velocity and directional persistence in comparison to SC motion on flat substrates. In addition, feature dimension affected SC morphology, alignment, and directional persistence. Plateaus and groove floors presented distinct cues which promoted differential motility and variable interaction with the topographical features. SC on the plateau surfaces tended to have persistent interactions with the edge topography, while SC on the groove floors tended to have infrequent contact with the corners and walls. Our observations suggest the capacity of SC to be guided without continuous contact with a topographical cue. SC exhibited a range of distinct motile morphologies, characterized by their symmetry and number of extensions. Across all conditions, SC with a single extension traveled significantly faster than cells with more or no extensions. We conclude that SC motility is complex, where persistent motion requires cellular asymmetry, and that anisotropic topography with cellular scale features can direct SC motility.

## Introduction

Studies of cellular motility inform a range of fields, from cell biology and pathology to biomedical and tissue engineering. Improperly regulated motility underlies cellular invasiveness in dieases such as cancer, and motile cells play vital roles in development, adult neurogenesis, wound healing, and homeostasis [1], [2]. Motility studies in vertebrate cell types have primarily focused on fibroblasts, neutrophils, and keratocytes, and this research has revealed a tightly regulated actin-based motility mechanism that varies with cell type and properties of the external cellular environment [3], [4]. Directed cell motility research has focused primarily on chemotaxis [5] and more recently, durotaxis [6]–[8], and has been conceptualized as a superposition of intrinsic cell directionality and influences from the external environment [9]. The current model of persistent cell migration describes a cycle of adhesion at a cell's leading edge, whole cell contraction, and rear-end retraction [10]–[12]; however, it is not clear how this model applies to symmetric cell types. Furthermore, cell speed, distance traveled, and direction of travel depend on many factors, including growth factors [13], extracellular matrix (ECM) components [13], [14], cellular morphology [15], [16], and extracellular topography [17]–[20].

An important aim of cellular and tissue engineering is the manipulation of cellular functions, including proliferation, differentiation, secretory behavior, cell-cell interactions, and motility, through tailoring the materials upon which cells are grown [21]. Contact guidance describes the phenomenon by which cells respond to extracellular topography by changing their morphology, alignment, and/or motility [22], [23]. The mechanisms by which these responses occur are not completely understood. A wide range of surface topographies have been shown to elicit contact guidance, including pillars and holes [24], [25], biomimetic features [26], aligned fibrils [27], and alternating grooves and ridges [28], [29]. Though some studies with larger features have been performed, the majority of studies with microgrooved substrates have presented grooves with spacing and depth on the order of the size of a cell body or smaller, ranging from 0.1 to 10 µm [30]–[33].

Attention has recently focused on the motility of Schwann cells (SC), the principal glia of the peripheral nervous system (PNS) [34]–[36]. During development, SC migrate ahead of and along axonal tracts, ensheath several axons and eventually segregate to wrap around a single axonal segment [37]. In response to PNS injuries, SC proliferate, produce growth factors, remove debris, and lay down longitudinal tracks that provide guidance and support for regrowing axons [38], [39]. Additionally, aligned live SC and isolated SC topography have the capacity to direct axon growth in vitro [26], [40], [41]. Because of their instrumental role in aiding PNS injury repair, SC have received attention as an important cell type with therapeutic applications. It has been recently suggested that therapies to promote nerve repair, notably nerve guidance channels, will require transplantation of supportive cells such as SC [42]–[44].

SC morphology makes them an interesting cell type for motility studies. SC tend to exhibit a bipolar morphology in culture, in which a long, spindle shaped soma is flanked by two thin extensions. A recent study found that SC in co-culture with neurons exhibited axon associated motility, which resulted from unipolar SC migration, while the bipolar SC phenotype was stationary [15].

Engineered microenvironments allow examination of SC response to their immediate environment and to substrates with potential therapeutic benefits. In this study, we examined SC morphology and motility in response to both flat control surfaces and culture platforms with grooves and plateaus tens of microns in width and depth. We evaluated the response of SC to this microgrooved topography by tracking SC movement over time, and quantifying their alignment, velocity and directional and turning behaviors. In addition, we characterized interactions between cellular components of SC and adjacent topographical features. Last, we examined the relationship between SC morphology and motile behaviors, characterizing the complex morphologies exhibited by migratory SC.

## Results

### SC migration was directed by anisotropic topography

#### Alignment

**Figure 1:**
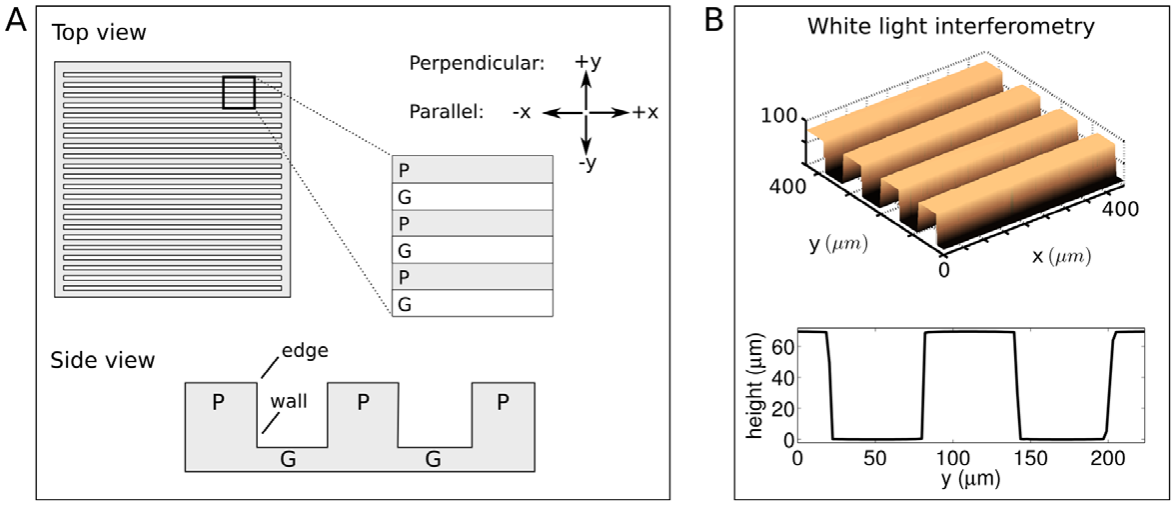
Experimental Setup. A. Top and side views of culture platforms, which consist of alternating raised (plateau, P) and indented (groove, G) regions. Arrows indicate directionality in motility studies: movement parallel to the topography occurs along the 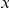 axis, and movement perpendicular to the topography occurs along the 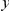 axis. Cells on plateaus encounter edge topography, and cells on grooves encounter corner and wall topography. B. 3D and profile views generated by WLI of a 60 µm-depth culture platform.

Cells were cultured on either flat PDMS substrate controls, or on microgrooved PDMS substrates which presented either an edge feature (for cells on the plateau level, “P”) or a wall feature (for cells on the groove floor, “G”). For the microgrooved substrates, the distance between walls or edges, referred to as the feature size, was either 30 or 60 m, resulting in four corresponding conditions referred to as P30, P60, G30 and G60. An overview of the experimental setup is shown in Figure 1A. While a plateau presented an edge and a descending wall, over which SC extensions could reach, a groove presented a corner and a wall, upon which SC extensions could ascend. White light interferometry (WLI) showed that platforms were rectangular and presented defined, repeating features (Figure 1B). High resolution WLI scans revealed that the plateau and groove surfaces had a slight curvature. The radius of curvature was 4.2
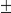
4.8 mm when these surfaces were approximated as circular.

**Figure 2:**
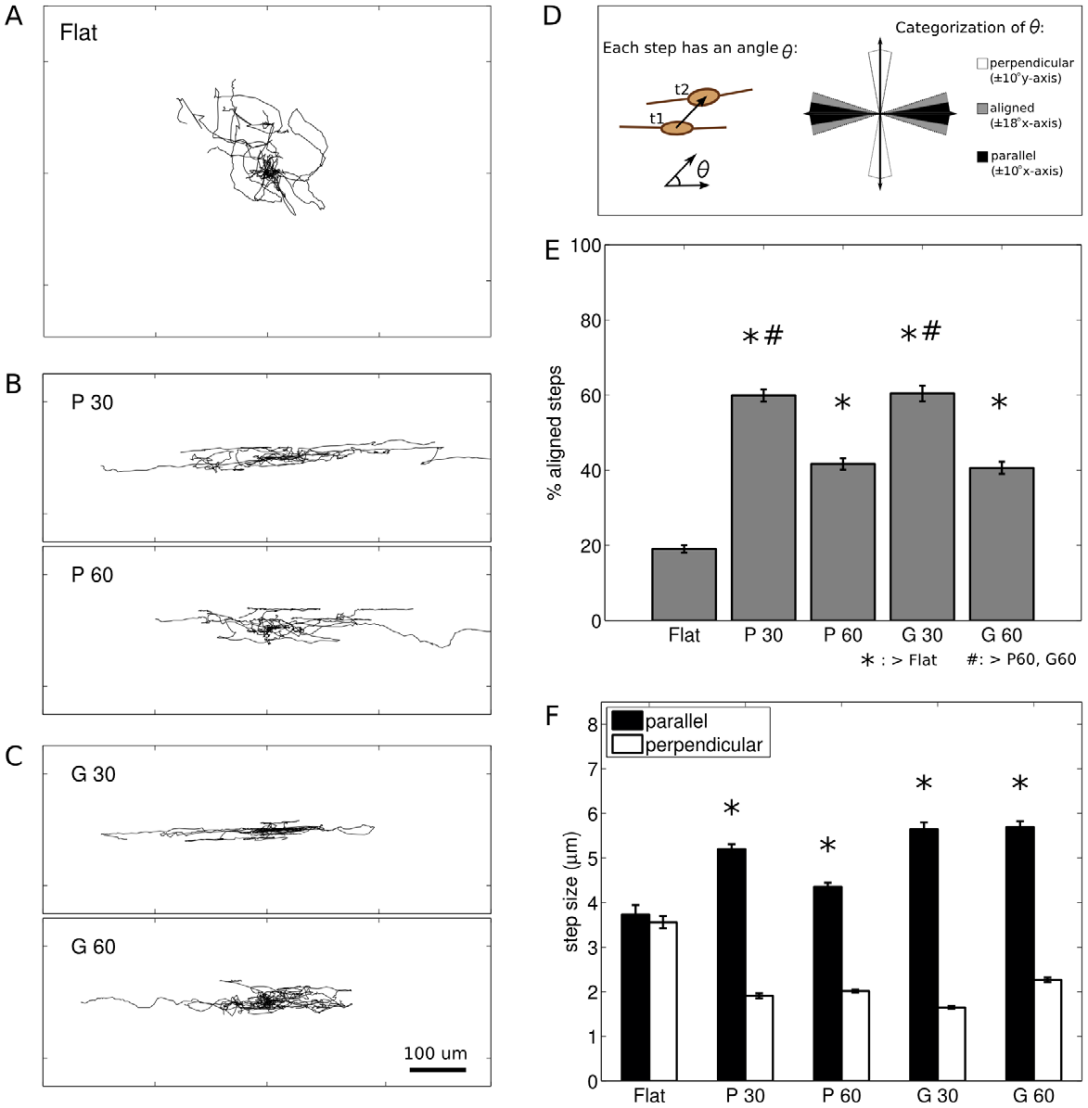
SC exhibited directed movement on anisotropic topography. A–C: Overlaid trajectories of 10 randomly chosen cells for each condition, normalized to start at 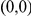. Scale bar is 100 m, and applies to all trajectories. A. SC on flat migrated randomly in all directions. B. SC on grooves migrated parallel to the direction of the topography. Top, 30 m grooves (G30). Bottom, 60 µm grooves (G60). C. SC on plateaus migrated parallel to the direction of the topography. Top, 30 m plateaus (P30). Bottom, 60 µm plateaus (P60). D. Cartoon shows method of categorizing the angle associated with each SC step into aligned, parallel, or perpendicular. E. Comparison of aligned movement between conditions. SC on all anisotropic conditions exhibited movement that was significantly more aligned than that found on flat. SC on P30 and G30 exhibited movement that was significantly more aligned than that found on P60 or G60. *,#: 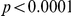, one-way ANOVA followed by post-hoc multiple comparisons with Sidak correction. F. SC steps 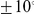 parallel to the topography were significantly larger than steps 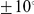 perpendicular to the topography. *: 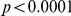, Mann-Whitney U between parallel and perpendicular. Error bars are SEM.

SC on flat controls migrated in all directions, with no overall directional preference, while SC on microgrooved substrates migrated parallel to the direction of the topography. Representative trajectories are shown in Figure 2A–C. Qualitatively, most cells, whether on flat or microgrooved substrates, exhibited locally persistent motion for some portion of their trajectories. To quantify the orientation of SC movement, the percent of the total steps each cell took within 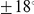 relative to the direction of the topography was determined, where a step is the movement of an individual cell in the interval 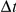 between sequential images (Figure 2D). For random motion with an arbitrary reference direction, the percent of aligned steps would theoretically be 20%. On flat substrates, 19.0
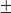
7.0% of SC steps were within 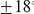 of an arbitrarily defined direction.

SC on anisotropic topography exhibited significantly more directed movement than SC on flat substrates (Figure 2E, Table S1). In particular, for SC on P60 and G60, 41.7
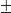
12.0% and 40.6
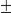
13.4% of steps, respectively, were aligned; and for SC on P30 and G30, 59.9
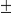
9.1% and 60.5
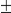
13.2% of steps, respectively, were aligned. The degree of aligned movement was affected by feature dimension, with the percentage of aligned steps significantly greater on the 30 µm platforms compared to the 60 µm platforms (Figure 2E). In addition, on microgrooved substrates the SC steps which were aligned within 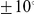 parallel to the feature axis were significantly larger than the steps which were oriented within 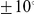 perpendicular to the feature axis (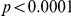, Mann-Whitney U, Figure 2F).

#### Velocity

**Figure 3:**
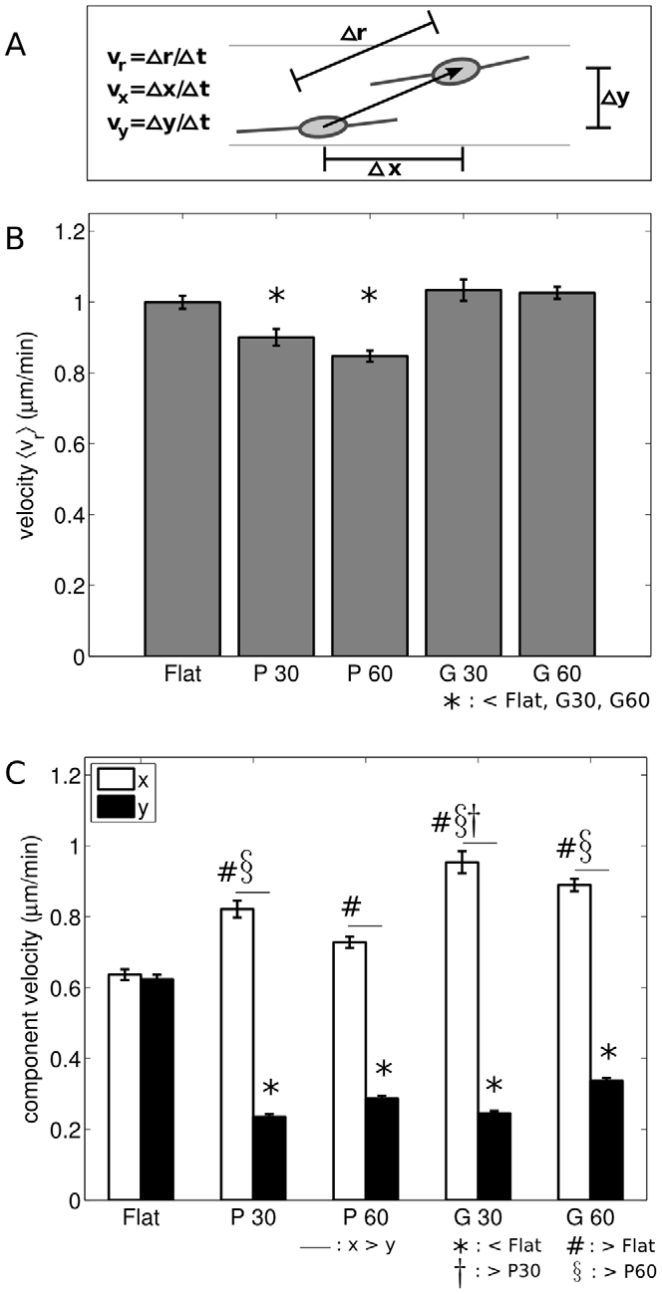
SC velocity was affected by anisotropic topography. A. Cartoon illustrating the calculation of overall velocity 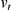, and parallel and perpendicular velocity components, 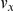 and 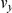, respectively. B. Graph of overall steady state velocity of SC cultured on flat and microgrooved substrates. C. Parallel (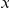) and perpendicular (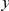) components of SC velocity on flat and microgrooved substrates.

For all conditions, the overall velocity of SC, 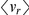 (Figure 3A), increased significantly over time (t-test between the initial two hours and final two hours, 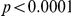). An apparent steady state velocity was reached at approximately 8 hours post-plating for all conditions. The steady state velocities of SC on the plateau conditions, P30 and P60 (0.90
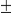
0.18 and 0.85
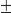
0.12 m/min, respectively), were significantly lower than SC velocity on flat (1.00
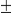
0.14 m/min), G30 (1.03
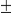
0.23 m/min), and G60 (1.03
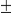
0.13 m/min) as shown in Figure 3B.

For SC on all microgrooved substrates, the steady state velocity parallel to the feature, 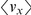, was higher than perpendicular to the feature, 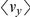 (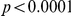, t-test, Figure 3C, Table S2). There was no difference between 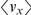 (0.64
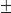
0.12 m/min) and 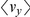 (0.62
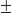
0.10 m/min) for the flat condition (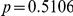) where the 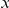 direction was chosen randomly and the 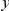 direction was orthogonal to 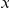. Therefore the velocity components of SC on flat platforms were pooled for comparisons with the microgrooved conditions, and are referred to as 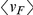.

The parallel component of velocity 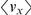 of SC on each microgrooved substrate was higher than 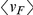 of SC on flat substrates (Figure 3C). In particular, while 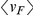 was 0.63
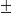
0.11 m/min for SC on flat platforms, 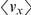 was 0.82
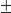
0.18 m/min (P30) and 0.73
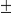
0.12 m/min (P60) for SC on plateaus and 0.95
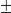
0.24 m/min (G30) and 0.89
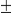
0.14 m/min (G60) for SC on grooves. Similarly, 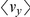 of SC on each microgrooved substrate was significantly lower than 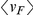 of SC on flat platforms, where 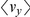 values for SC on anisotropic conditions were 0.24
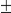
0.06 m/min (P30), 0.29
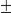
0.06 m/min (P60), 0.25
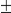
0.05 m/min (G30), and 0.34
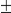
0.06 m/min (G60). In addition, 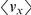 was also significantly higher on G60 than P60, and on G30 than P30 (Figure 3C, Table S2).

#### Directional and Turning Behaviors

**Figure 4:**
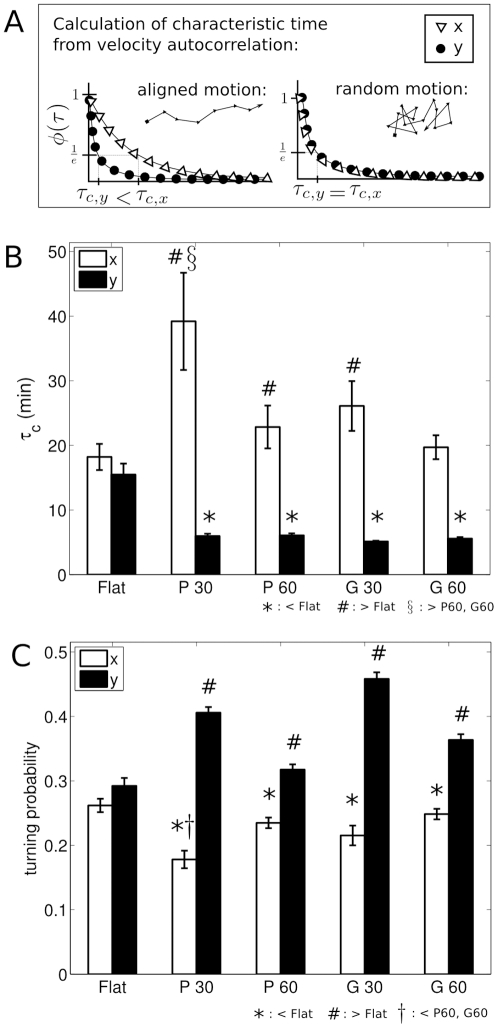
SC directionality was stronger on anisotropic than flat substrates, as measured by velocity autocorrelation and turning probabilities. A. Cartoon demonstrating the calculation of characteristic drop off times, 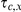 and 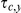, from the autocorrelation 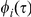, based on theoretical aligned and unaligned motion. B. Characteristic drop off times, 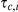 calculated from the velocity autocorrelation 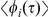 for each condition and direction. *,#: significant in Mann-Whitney U tests against 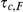. 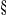: significant in multiple comparisons across anisotropic conditions with Sidak correction. C. Graph shows turning probability parallel (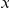) and perpendicular (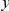) to the topography. *, #: significant in pairwise t-test against flat overall turning probability. 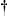: significant in multiple comparisons across anisotropic conditions with Sidak correction.

In order to assess the directional behavior of SC on flat and anisotropic substrates, the velocity autocorrelations 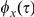 and 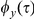 were calculated based on 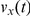 and 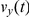 for each cell. Velocity autocorrelation for an individual cell was calculated using the following equation:
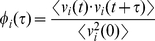
(1)Where 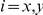 and here the angle brackets indicate that this operation is calculated over all 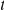 for a given 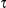, where 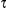 is the lag between time points used for the autocorrelation calculation. SC velocity autocorrelation decreased exponentially over time, and was described by a characteristic time constant, 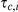, for each experimental condition and direction. The time constant 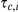 is the time at which 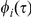 has dropped to 1/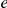 (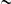
37%). As indicated in the cartoon (Figure 4A), persistent directional motion will yield a larger value of 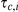 than random or antiparallel motion.

For SC on flat substrates, 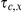 was not significantly different from 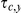 (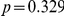, Mann-Whitney U), where the directions were as described above. The pooled data is referred to as 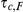, and was 16.84
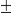
13.62 min. In pairwise comparisons against 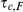, SC on the P30, G30, and P60 substrates had significantly higher average values of parallel characteristic time, 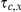 (Figure 4B, Table S3). SC on the G60 substrates followed the trend of increased 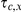 when compared to 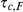, but did not reach statistical significance (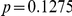, Mann-Whitney U). Further, SC on all anisotropic conditions exhibited a significantly lower perpendicular characteristic time, 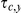, when compared to SC on flat (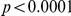, Mann-Whitney U). SC directional persistence parallel to P30 as measured by 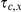 was significantly higher than that of P60 or G60 (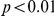 and 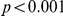, respectively, Mann-Whitney U followed by Sidak correction for multiple comparisons).

The average values of 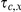 ranged from approximately 20 to 40 minutes, indicating that after these time periods, SC movement in the 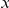 direction was 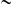
37% correlated with its initial speed and direction (Figure 4B). Thus, 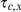 is persistence time parallel to the topography. On flat controls, 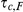 was approximately 17 minutes, which indicates that for a random direction, SC movement was 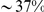 correlated with its initial speed and direction after 17 minutes. Together, these results indicate that SC on anisotropic substrates continued to move parallel to the features for a longer time than they would have continued in a given direction on a flat material. The relatively low average values of 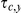 for SC on anisotropic subtrates ranged from 5 to 6 minutes, indicating that SC frequently changed their orientation perpendicular to the feature (Figure 4B). These times are on the order of 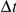, the time between sequential images in the timelapse recordings. Because values of 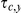 were close to the low end of the temporal resolution of this experiment, it is possible that the SC were changing orientation in the perpendicular direction more frequently than was detected by this analysis.

To further quantify the directional behavior of SC on flat and anisotropic substrates, turning probabilities were calculated for the 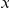 and 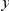 directions (Figure 4C, Table S4). For SC on flat substrates, the parallel turning probability was not significantly different than the perpendicular turning probability (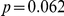, t-test) where the directions were as described above. The pooled data is referred to as the overall turning probability on flat, and was 0.28
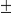
0.084.

A lower 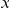 direction turning probability relative to that found for SC on flat substrates indicates a higher relative parallel directional persistence. Conversely, a higher 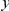 direction turning probability indicates a lower perpendicular directional persistence. There were significantly lower values of 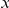 direction turning probabilities for SC on all anisotropic substrates when compared to the turning probabilities for SC on flat substrates (Figure 4C, 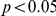, pairwise t-tests). Similarly, there were significantly higher values of 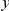 direction turning probabilities for SC on all anisotropic substrates when compared to the turning probabilities for SC on flat substrates (Figure 4C, 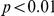, pairwise t-tests). In comparisons of 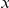 and 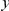 turning probabilities, SC on all anisotropic substrates had significantly lower average parallel turning probabilities compared to the corresponding perpendicular turning probability (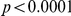 all conditions, t-test).

Lastly, there was a trend toward higher directional persistence parallel to the topography when comparing SC on 30 m platforms to SC on 60 m platforms. The SC turning probability in the parallel direction was significantly lower on P30 when compared to P60 and G60 (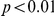 and 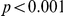, respectively, post-hoc pairwise t-test across anisotropic conditions with Sidak correction for multiple comparisons).

### Variable contact with features resulted in aligned motility

**Figure 5:**
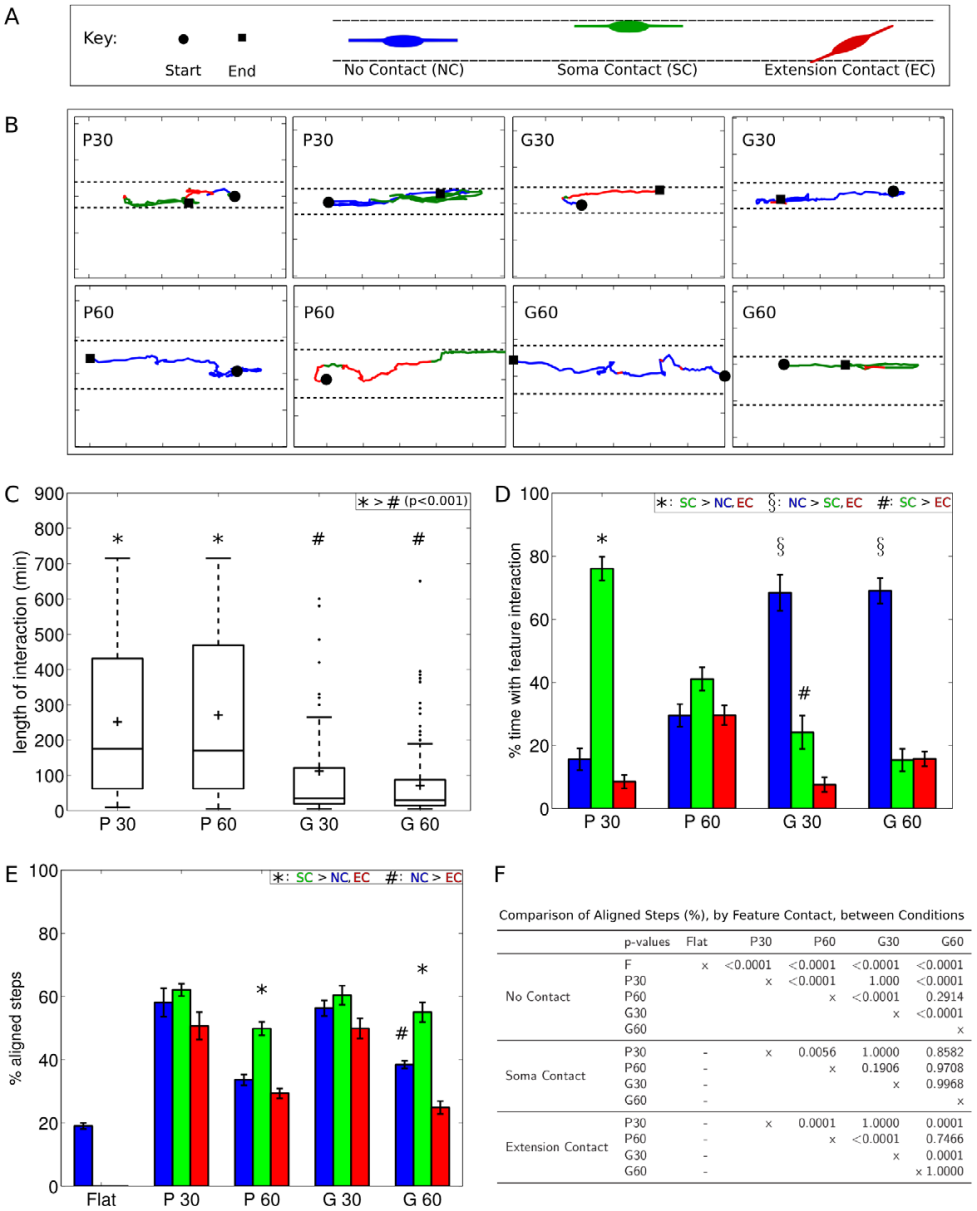
Aligned SC movement correlated with variable contact between SC and topography. A. Illustration of how SC contacted the feature through their soma (green) or extension(s) (red), and when SC had no visible contact with the feature (blue). B. Representative trajectories of two randomly selected cells from each condition show SC movement when SC contacted the feature through their soma (green) or extension(s) (red), and when SC had no visible contact with the feature (blue). Tick marks are spaced 50 m apart. Dashed lines indicate the location of the edges and walls of the plateaus and grooves, respectively. C. SC on plateaus and grooves exhibited variable lengths of time of continuous contact with features. Boxplot shows lengths of time during which SC continuously contacted a feature. ‘+’ indicates mean interaction times, tops and bottoms of boxes indicate upper and lower quartiles, bands indicate medians, whiskers indicate minimum and maximum values, and dots indicate outliers. *,#: significant in Kruskal-Wallis ANOVA followed by multiple Mann-Whitney U comparisons with Sidak correction. D. Bar graph of percentage of total trajectory spent with each type of contact between SC and the features. *,#,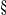: significant in comparisons within the conditions for % time spent with no contact, soma contact, or extension contact, by post-hoc multiple comparisons with the Sidak correction. E. Bar graph of percentage of aligned steps in each condition, categorized by type of contact between SC and the features. *,#: significant in comparisons within the conditions for % alignment based on feature contact, by post-hoc multiple comparisons with the Sidak correction. F. Table shows p-values for comparisons across the conditions for % alignment based on feature contact. Following an ANOVA (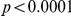), post-hoc multiple comparisons with the Sidak correction were performed. Error bars are SEM. A black and white version of this figure is available, Figure S1.

Interaction between cellular features (soma and extensions) and topographical features varied over time (Figure 5, S1). As SC migrated on the plateaus or grooves, they had either no contact with a wall or edge, or contact by either the cell soma or at least one cellular extension, as illustrated in Figure 5A. Two representative trajectories from each condition are shown in Figure 5B, and have been color-coded based on the observed contact between cellular features and topographical features. As indicated by these trajectories, many periods of aligned movement occurred without visible contact with features (blue lines). In other cases, SC somas (green lines) or extensions (red lines) contacted the topographical features for periods of time.

The length of time SC continuously contacted the features was quantified (Figure 5C). SC on P30 and P60 continuously contacted the plateau edges for median times of 175 and 170 min, respectively. SC on G30 and G60 continuously contacted the groove walls for median times of 35 and 30 min, respectively, which were both significantly shorter periods of time compared with the continuous interaction times found for SC on the plateau conditions. The amount of time SC in each condition spent with no contact, soma contact, or extension contact is shown in Figure 5D. Comparisons within each condition show that on P30 and P60, SC followed a trend towards spending more time with soma-feature contact than no contact or extension contact (Table S5). In contrast, SC on G30 and G60 spent the majority of the time with no feature contact.

For each type of contact, the percentage of steps aligned within 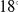 of the feature direction was calculated (Figure 5E). Within each condition, aligned movement was highest when SC soma were in contact with the feature (Table S6). For the P60 and G60 substrates, the aligned movement in the soma contact condition was significantly higher than the aligned movement in the no contact and extension contact conditions.

When SC had no visible contact with the features (Figure 5E, blue bars), all conditions exhibited significantly higher aligned movement than was found on flat substrates (Figure 5F, top). Furthermore, both 30 m conditions had significantly higher aligned movement than the 60 m conditions. When SC had visible contact between the soma and the feature (Figure 5E, green bars), there was a trend towards higher alignment on the 30 m plateau substrates compared to the 60 m conditions (Figure 5F, middle). When SC had visible contact between at least one cellular extension and the feature (Figure 5E, red bars), SC on the 30 m substrates had significantly higher aligned movement than SC on the 60 m substrates (Figure 5F, bottom).

### Motile SC morphology was highly variable; unipolar cells moved fastest

**Figure 6:**
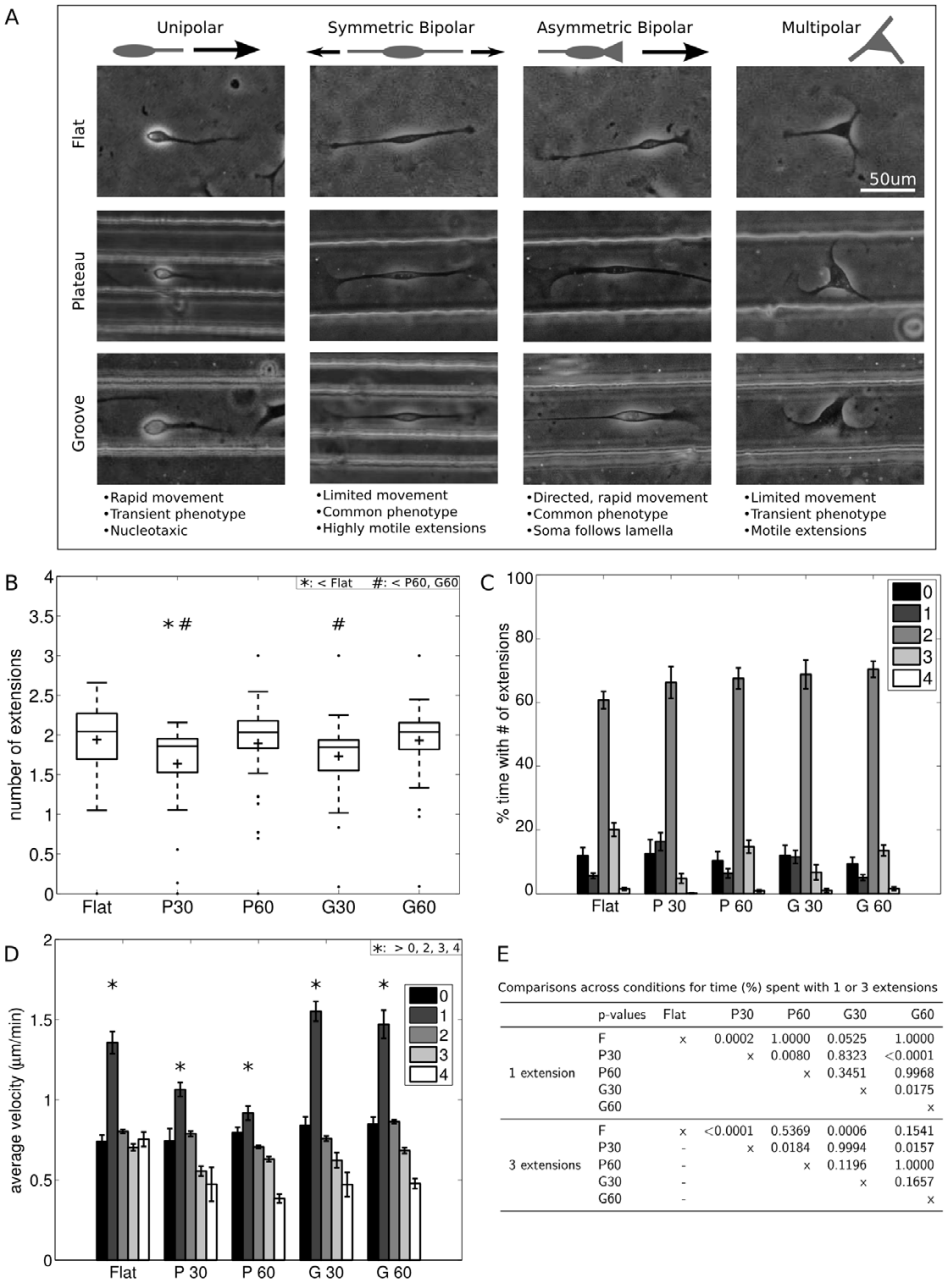
SC exhibited multiple distinct motile morphologies. A. Phase contrast micrographs show SC with unipolar, bipolar, and multipolar morphologies. B. Boxplot shows number of extensions per cell on each substrate, where the distribution varied with feature dimensions. ‘+’ indicates mean number of extensions. Tops and bottoms of boxes indicate upper and lower quartiles, bands indicates medians, whiskers indicate minimum and maximum values, and dots indicate outliers. *,#: significant in comparisons between conditions for number of extensions, by post-hoc multiple comparisons with the Sidak correction. C. Amount of time SC on each substrate spent with zero through four or more extensions. D. SC velocity correlated with number of extensions. Graph shows SC velocity as a function of the number of extensions (0–4). For all conditions, SC exhibiting one extension (unipolar morphology) migrated with a significantly higher velocity than SC with zero, two, three, or four extensions, indicated with ‘*’. E. Table shows p-values from comparisons across conditions for % time spent with 1 or 3 extensions. Following an ANOVA (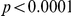), post-hoc multiple comparisons with the Sidak correction were performed. Error bars are SEM.

SC exhibited a number of distinct motile phenotypes (Figure 6A). In one mode, SC tended to be symmetric and bipolar (Figure 6A, second column), though they were occasionally multipolar (Figure 6A, fourth column), and spent the majority of their time moving the tips of their extensions as the soma sampled the local space. Qualitatively, the symmetric bipolar and multipolar phenotypes involved exploration and searching, and were not correlated with an overall directedness. Another common SC morphology resembled that of migrating fibroblasts, where SC were asymmetric (Figure 6A, third column), with one relatively flat and wide extension (leading edge) as well as a second relatively long and thin extension (trailing end). SC with this morphology moved in the direction of the leading edge. The soma and extensions were generally distinct structures, with the leading edge being similar in appearance to a neuronal growth cone or lamella, and the overall shape of the SC elongated and bipolar. In addition, SC occasionally exhibited a keratocyte-like motion, in which the cell soma was elongated with two thin phase-dark extensions emanating from the soma, and migrated in the direction perpendicular to the cell's long axis (no image shown). Last, some motile SC exhibited a unipolar phenotype (Figure 6A, first column). This phenotype was transient, and followed a particular sequence of events: a bipolar SC would retract one of its extensions into its soma, followed by a quick movement of the soma towards the anchored tip of the remaining extension, thus covering a relatively large distance in a relatively short period of time. From here, SC would temporarily be relatively stationary while re-establishing a bipolar morphology.

These observed SC phenotypes were quantified in terms of the number of extensions at each time point during the timelapse recording (Figure 6B). SC on 30 m features had significantly lower average number of extensions compared to SC on the flat and 60 m conditions (Table S7). During the recording, SC on flat had an average of 1.94
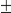
0.48 extensions, and SC on P60 and G60 had averages of 1.89
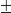
0.53 and 1.93
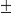
0.42 extensions, respectively, while SC on P30 and G30 had significantly fewer extensions, with averages of 1.64
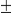
0.54 and 1.73
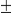
0.44, respectively. Examination of the distributions of the average number of extensions for each cell shows that SC on flat substrates were normally distributed (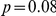, Lilliefors test for normality). SC on P60 and G60 had similarly shaped distributions of extensions per cell, while SC on P30 and G30 also had similarly shaped distributions, none of which were normally distributed (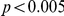, Lilliefors test).

The percent of total time that SC spent with each number of extensions was calculated for each cell (Figure 6C). SC in all conditions spent the majority of the time with two extensions, ranging from 60.7
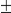
19.8% on flat to 70.4
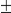
20.7% on G60 (no significant difference between conditions, 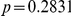, ANOVA). SC on P30 exhibited significantly more time with one extension (16.34
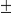
15.5%) compared with SC on P60 (6.40
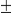
11.8%). Similarly, SC on G30 exhibited significantly more time with one extension (11.53
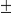
13.4%) compared with SC on G60 (5.05
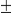
7.6%). Further, SC on P30 and G30 exhibited significantly less time with three extensions (4.8
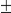
8.3%, 6.7
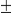
15.1%) when compared with SC on flat (20.08
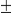
15.5%), 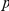-values from comparisions between conditions for time spent with one or three extensions shown in Figure 6E. There were no significant differences between the amount of time spent with zero or four extensions.

There was an inverse relationship between the number of SC extensions and velocity, with a significant correlation (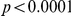) between the number of extensions and velocity for SC on all substrates tested. Across all conditions and time points, SC with a single extension migrated with a significantly higher velocity, compared to SC exhibiting fewer or greater numbers of extensions (Figure 6D, 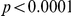 for flat, P30, G60; 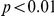 for G30; 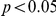 for P60; Mann-Whitney U comparing velocity by number of extensions for each condition, followed by Sidak correction for multiple comparisons). The unipolar morphology was relatively rare, comprising only 5–15% of a cell's trajectory (Figure 6C). Median times for continuous unipolar migration were 15–30 minutes in length, indicating that the unipolar morphology was relatively transient.

**Figure 7:**
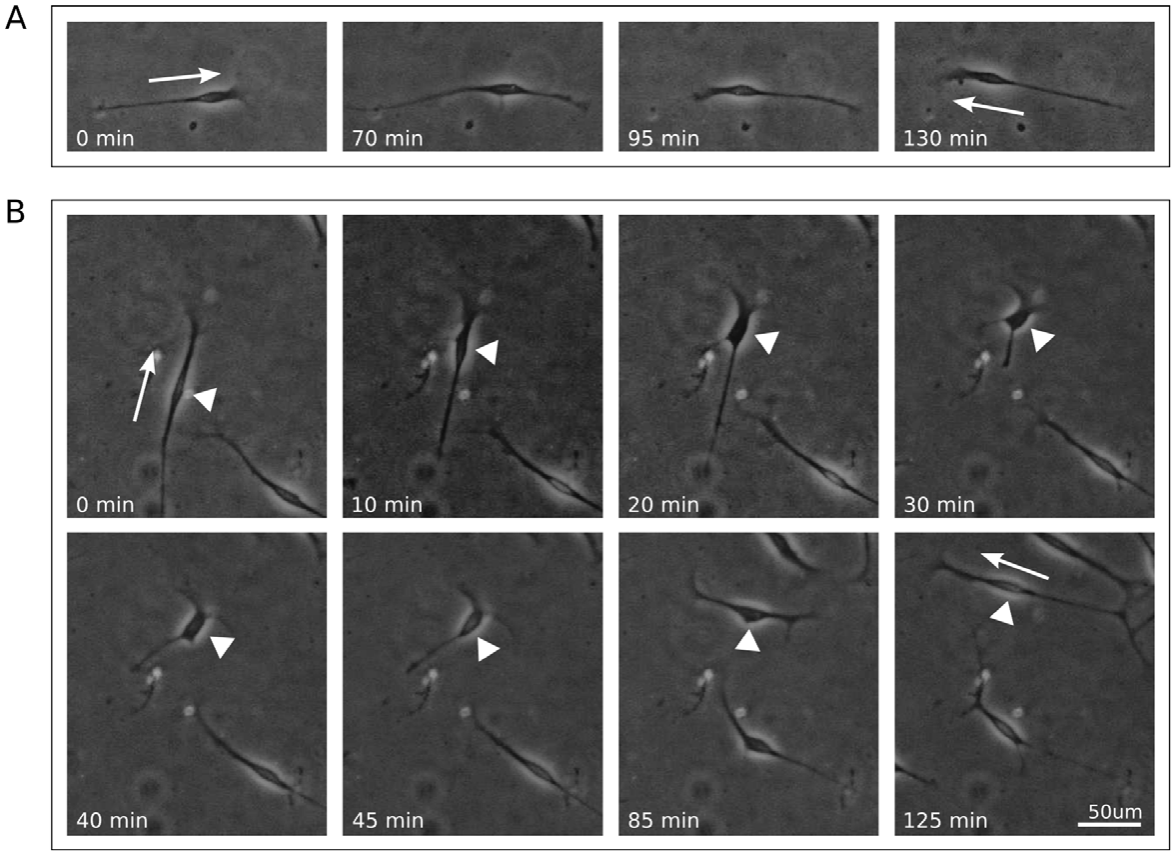
SC changed direction in two ways. A. SC on flat substrate reversed direction through switching its leading and trailing edges. B. SC on flat substrate changed direction to be perpendicular to the original direction. A,B: Arrow indicates initial and final directions of movement. B: Arrowheads point to soma of interest. For each image sequence, the field of view shown is identical.

On flat substrates, SC changed direction in two ways (Figure 7). In the first type of directional change, migrating SC with an asymmetric bipolar morphology paused and the leading and trailing extensions subsequently switched functions (Figure 7A). In the second, more complex, turning behavior, the leading extension paused while the soma continued forward to join it, and the trailing edge retracted. Simultaneously, the leading edge continued to spread out, elongating in the direction perpendicular to the previous direction of movement. In this way the SC turned 90°, and thus began to migrate perpendicular to its original direction of motion (Figure 7B). Often, many of these transitions followed each other in rapid succession, resulting in frequent directional changes and little overall displacement.

When SC were cultured on microgrooved platforms, the overall migration characteristics were similar to those observed on flat controls. However, the most common motile SC morphologies seen on these surfaces were asymmetric and bipolar, characterized by a correspondence between the direction of cellular alignment and the direction of cellular migration. In addition, the turning behavior described above (Figure 7B) which resulted in perpendicular directional change was not observed for SC on microgrooved platforms.

## Discussion

The main objective of this study was to compare the motility of SC on materials presenting no topographical cues to their motility on materials presenting well-defined anisotropic topographical cues. Toward this end, we chose materials with alternating grooves and plateaus presenting sharp wall and edge features, respectively, that were separated by distances on the order of or larger than the SC cell body. We hypothesized that in comparison with isotropic materials, anisotropic topography would alter SC migration, and that both feature type and dimension would have distinct effects on SC motility. Comparisons of motility parameters derived from SC trajectories on flat and anisotropic materials were used to test this hypothesis. In addition, we described the motility of SC in terms of number of extensions, overall shape, and interaction with topographical features. We found that SC on microgrooved surfaces migrated parallel to the direction of the topography, with variation in migratory characteristics that depended on feature width and type. We also found that SC motility was complex and an individual SC could exhibit multiple distinct motile phenotypes, with a rare unipolar morphology being correlated with a significantly increased velocity.

For both 30 and 60 m conditions used in this study, the distance between features was larger than the cell body width of a typical oriented SC, which has been previously measured to be approximately oval in shape with a major axis of 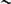
36 µm and a minor axis of 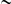
13 µm [45]. SC soma, extensions, and overall movement were preferentially aligned parallel to the topography. These results are consistent with those reported previously for fibroblasts, endothelial cells, and smooth muscle cells, where migration was directed parallel to subcellular sized grooves [46]. In addition, aligned steps were approximately three times larger than steps oriented perpendicular to the topography.

Overall SC velocity was significantly lower on plateau substrates relative to flat with a relative difference of 10–15%, whereas SC velocity on groove substrates had a non-significant trend towards a higher velocity relative to flat controls. In addition, the parallel direction velocity 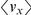 was higher for SC on microgrooved substrates than the equivalent velocity component of SC on flat controls, while the reverse effect held for the perpendicular direction velocity 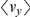. In particular, 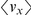 of SC on microgrooved substrates was up to 50% higher than for SC on flat controls. Similarly, 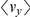 of SC on microgrooved substrates was up to 60% lower than for SC on flat controls. Therefore, the observed effect of cellular-scale plateau and groove topography was the orientation of SC movement parallel to the direction of the topography, while overall SC velocity was relatively similar regardless of topographical cue. Previous studies that evaluated cellular velocity on grooved surfaces have found mixed results. For example, subcellular-sized grooves have been shown to increase the migration speed of fibroblasts [47], reduce the migration speed of PC12 cells [48], and to have varied effects on the migration speed of fibroblasts depending on specific feature sizes [49], [50].

SC changed their migration patterns in response to microgrooved substrates by moving parallel to the direction of the topography, as indicated quantitatively by the characteristic time constant of their directional velocity autocorrelation and their turning probability. It is possible to imagine that when confronted with barriers to migration on two sides, SC would significantly reduce their overall movement rather than orienting to the topography. However, SC movement was aligned on microgrooved substrates, resulting in parallel direction migration. Persistence time is inversely proportional to a cell's tendency to change directions and is affected by cues from the environment [2], [13], [14]. Rather than evaluating a two-dimensional persistence time, we analyzed the parallel and perpendicular components of SC movement separately. For the component of SC movement parallel to the topography, there was a trend towards increased velocity autocorrelation and decreased turning rate, indicating that SC confronted with cellular-scale anisotropic topographical cues tended to move parallel to the feature, and to continue in this fashion for longer than they would have with no cue. In contrast, for the component of SC movement perpendicular to the topography, there was a trend towards decreased velocity autocorrelation and increased turning rate, indicating that SC oscillated perpendicular to the channels at a relatively high rate, at least on the order of the experimental sampling rate of five minutes. We hypothesize that SC respond to topography by orienting without altering their motility machinery, thus resulting in cell movement which is directional but not fundamentally altered in its mechanism, including actin-based cytoskeletal dynamics. This would explain the relatively small difference between overall velocities of SC on flat and microgrooved conditions.

SC responded differently to plateaus and grooves in terms of feature contact and motility characterisitcs. In particular, SC on grooves traveled faster than those on plateaus. Further, SC on plateaus spent significantly more time contacting the feature edges than SC on grooves spent contacting the feature walls. This may indicate that an overall increase in contact between SC and topographical features reduces overall motility. One caveat to these observations is the slight curvature, whose radius was measured to be approximately 4 mm, of the groove floors and plateau surfaces. Luminal curvature can result in alignment of growing neurites, though this type of effect has been shown to be essentially nonexistent for fibers with a 250 µm or larger radius [51], so it is unclear what role curvature played here. Both raised and indented features have been studied, and it has been suggested that the mechanism of guidance at play differs when cells see indented versus raised features [17], [52]. These results imply that when designing guiding biomaterials for specific applications, it may be necessary to consider not only the dimension of the features presented but also their particular geometry.

The distance between features was important in determining the response of SC to microgrooved surfaces. SC alignment, number of extensions, and directional behavior, were all affected by the 30 µm conditions, compared to the flat or 60 µm conditions. SC responded to narrow features with lower average numbers of extensions, a higher percentage of time with one visible extension, and a lower percentage of time with three visible extensions. Further, SC on the 30 µm materials exhibited higher aligned movement, higher parallel directional persistence, lower parallel direction turning rates, and higher perpendicular direction turning rates when compared to SC on the 60 µm materials. Together, these results indicate that a smaller distance between topographical features can reduce the prevalence of a multipolar morphology and result in movement that is more strongly oriented.

Our observations suggest the ability of SC to be guided without continuous contact with a topographical cue. During time points when SC had no visible contact with a feature they moved in a significantly aligned manner. Possible explanations for these unexpected observations include the following: (1) SC are getting physical feedback from the topography through (a) contact that was brief and occurred in the time between images, and/or (b) contact with SC extensions which were too thin to appear in the phase contrast micrographs; (2) SC are responding to the slight curvature of the surfaces; and (3) SC did not require consistent contact with directive cues because variable or infrequent contact oriented the cells, and the resulting polarity manifested in persistently aligned motion. Further study will be required to determine the potential contribution of each of these mechanisms to the variable contact observed.

Last, SC exhibited complex motility, with multiple distinct motile morphologies. These were characterized qualitatively in terms of their symmetry, and quantitatively in terms of the number of extensions. SC typically have a distinct bipolar morphology in culture [53], in contrast to many cell types used in motility studies which present asymmetry corresponding to distinct leading and trailing edges. While symmetric, bipolar SC did exhibit movement, the soma tended to follow a localized random walk and the extension tips were highly motile. In contrast, asymmetric SC, both unipolar and bipolar, tended to exhibit more persistent motion. Further, there was a significant correlation between the number of extensions and the velocity of SC, both on flat and microgrooved materials. Previous studies have indicated that cellular morphology is causally linked to cellular motility [54], [55].

SC were observed to change their direction through two distinct methods. These involved a simple reversal of polarity, and a more complex orthogonal reorientation. In contrast to the polarity reversal observed for SC, neutrophils have been reported to maintain their leading edge, tracing out a “u-turn” if confronted with a sharp gradient reversal [56]. The SC turning behavior in which the leading and trailing extensions are retracted while new extensions are protruded may be similar to that found in fibroblasts, that turn by formation of a peripheral lamella which can become the dominant, leading edge [57].

Previous studies of cell-material interactions with groove spacing or depth larger than 10 m in dimension have found that features on the order of, or larger than, the size of a cell can promote cellular alignment parallel to the direction of the grooves. Mahoney et al. reported that PC12 pheochromocytoma cells aligned their soma and neurites on substrates with patterned polyimide walls 11 m high and 20–60 µm wide, with alignment proportional to proximity to the wall [29]. Similarly, Li and Folch found that neurites extended parallel to plateau edges if the height was on the order of or larger than the cell body, even if the angle of approach to the edge required a turn of up to 90° [58]. Examining the cellular dynamics underlying alignment, Tan and Saltzman found that neutrophils on surfaces with raised ridges had the highest motility when the ridges were separated by 10 µm, which is approximately the diameter of the cell body. However, when the spacing was wider, 12 or 14 µm, cells remained in constant contact with one wall and exhibited a reduction in motility compared to on the 10 µm spacing [17]. They also noted that membrane extensions may determine cell speed, observing that membrane protrusions were inhibited perpendicular to the topography, while they were favored parallel to the topography. Other than the information provided by these recent studies, little is known about the motility of cells on microgrooved substrates with dimensions larger than the cell body. Here we have presented a detailed mathematical analysis of cellular motility on large-scale anisotropic topography, coupled with qualitative descriptions of the cellular motility characteristics.

Contact guidance studies exploring the cytoskeleton have investigated features with subcellular dimensions. Alignment of cytoskeletal filaments and focal adhesions has been demonstrated to occur along the sharp edges of subcellular grooves [59]. It has been hypothesized that contact guidance to subcellular sized grooves results from mechanical stresses created by adhesions to the aligned ridges of the substrate [23], [59], [60]. Studies showing alignment of actin microfilaments and microtubules parallel to subcellular sized grooves support this hypothesis [28], [30], [59], [61]. One implication of this hypothesis is that multiple aligned contacts between the cell and the substrate are required for contact guidance. However, in the experimental setup used here, SC did not have numerous subcellular ridges to adhere to, and the relatively large feature scale resulted in the unique phenomenon of variable contact between SC and the features. Other mechanistic explanations for contact guidance include the hypothesis that topographical cues enhance localized adhesions and contractions [24], or that topography may trigger a range of intracellular pathways [62].

### Conclusions

This study provides an in-depth descriptive and mathematical analysis of SC motility on both flat and grooved surfaces. In comparing flat and anisotropic materials, we found stronger effects of topography on directional behavior than on the overall velocity. We propose that cellular response to topography is an active process, and is more complex than simply cytoskeletal restriction. We hypothesize that in the experiments presented here, the topography induces initial alignment through direct contact between SC and the feature. This initial orientation is continued by the SC, with occasional or periodic additional contact with the guiding feature, and thus the establishment of polarity may be a significant feature of the contact guidance mechanism at play here. These results suggest that type and dimension of topographies can affect the behavior of SC, and it will be interesting to determine whether similar mechanisms are at play in topography-induced directional persistence and in chemotactic guidance.

## Materials and Methods

### Microgrooved Substrate Fabrication

Microgrooved substrates were fabricated as previously described [63], [64]. Briefly, patterns were designed in AutoCAD (Autodesk), in which opaque and transparent regions were arrayed, with 30 or 60 m spacing, in 1 cm square sections. These patterns were printed on mylar films at 10,000 dpi (CAD/Art Services, Inc.) and used as a mask to generate selectively polymerized regions during the photolithography process to create alternating grooves and plateaus. Standard photolithographic techniques were used to fabricate substrates, in which silicon wafers were spin-coated with a uniform layer of negative tone Nano SU-8 50 photoresist (Microchem) to a height of 50 µm, according to the manufacturer's protocol (CEE100 spinner, Brewer Scientific). The photoresist was baked at 65

C for 6 min, and 95

C for 20 min. After cooling, the photoresist was selectively polymerized by UV exposure (365 nm) through the mask using a Karl Süss aligner, and post-exposure baked for the same times and temperatures as pre-exposure baking. Unpolymerized photoresist was washed away using SU-8 developer as recommended by the manufacturer until the features were resolved. The negative photoresist patterns contained protruding features, and were cast using poly(dimethyl siloxane) (PDMS, Sylgard 184 elastomer base and curing agent mixed at 10∶1 wt/wt), creating positive, indented surfaces. Flat controls were fabricated by casting PDMS onto tissue culture dishes (Fisher).

Substrate profiles were generated by white light interferometry (WLI). WLI data scans of PDMS substrates were recorded with a 20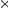 objective at 1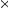 zoom with a Zygo New View 6000 3D profiler (Zygo Co., Middlefield, CT, USA) and analyzed using Gwyddion software (v2.0, Czech Metrology Institute, Brno, Czech Republic).

### Substrate Treatment

All coating and cell culture reagents were from Gibco unless stated otherwise. PDMS substrates were cut from the silicon wafers or tissue culture dishes into 1 cm squares, and activated for 1 min in a plasma etcher/sterilizer (PDC-32 G, Med RF level, Harrick). Platforms were immediately covered with 70% EtOH to be kept wet and sterile. Following two washes with Hank's Balanced Salt Solution (HBSS), platforms were coated with 0.01% poly-L-lysine (PLL, Sigma, St. Louis, MO) for 1 hr at room temperature (RT). Substrates were rinsed twice with HBSS, and coated with 50 g/mL laminin (LN) for 1 hr at RT.

### Cell Culture

Schwann cells isolated from adult rat sciatic nerve (SC, passage 4 through 10, a gift from Dr. Mary Bunge, University of Miami) were used in these studies. SC were maintained in SC media, Dulbecco's Modified Eagle's Medium (DMEM 11965) supplemented with 10% fetal bovine serum (FBS), 4 mM L-glutamine, 2 µM forskolin (Sigma), 10 g/mL bovine pituitary extract (Sigma), 226 M heregulin (a gift from Genentech), 100 U/mL penicillin, and 100 g/mL streptomycin. Prior to timelapse studies, cells were detached from tissue culture flasks with 0.25% trypsin-EDTA and resuspended in SC media to achieve a final density of 1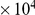 cells/cm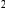. This relatively low cell density was used to reduce cell-cell interactions to facilitate the examination of individual SC morphology and motility.

### Timelapse Microscopy

Cells plated on microgrooved platforms were cultured at 37

C and 5% CO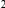 in a microscope incubation chamber. Images were taken with a Nikon Eclipse TE2000-S microscope with phase contrast optics, connected to a Hamamatsu Orca-ER camera, Orbit shutter controller (Improvision), and collected by OpenLab software (v4.0.4, Improvision). Fields of view were randomly chosen for each substrate, and a custom Openlab automation was used to record an image at each postion at an interval of 5 minutes over a period of 12 hr, starting 1 hr after cells were plated.

### Position and Attribute Tracking

Cell positions for at least 30 cells from at least three different fields of view from at least two independent experiments for each condition were tracked manually using a modified ImageJ (NIH) plugin. At each timepoint, the 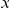 and 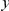 coordinates of the cellular position were recorded for each cell, as well as a classification of the cell morphology and associated interactions. Substrates were oriented so that SC movement parallel to the long axis of the topography was in the 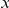 direction, and SC movement perpendicular to the long axis of the topography was in the 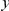 direction. These measurements included number of SC extensions (0–5) and visible interactions with substrate feature (no contact, soma contact, or extension contact). In addition, if SC were visible on the substrate wall, their movement was tracked but not their cellular morphology due to optical limitations. Data corresponding to SC movement on the wall were not used when analyzing parameters in terms of the morphological characterizations. Cell trajectories were normalized to start at 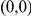 at the first time point. All computations were carried out in Matlab (v. 7.7, R2008b, The Mathworks).

### Motility Analysis

Percent alignment was calculated for each cell by determining the percentage of steps which were aligned within 18 of the direction of the topography. For SC on flat controls, percent alignment was calculated relative to an arbitrary direction. SC movement was binned by angle, and steps within 10 of the 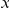 or 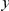 axis were considered parallel or perpendicular, respectively, and were compared to each other.

Average velocity was calculated for each cell between consecutive timepoints by the following formulas: overall velocity, 
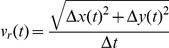
, parallel velocity, 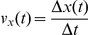, and perpendicular velocity, 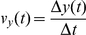. The absolute values of 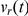, 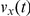 and 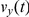 were averaged together at each timepoint for all cells in a given condition; this is indicated by 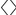.

Velocity autocorrelation was determined according to the formula 
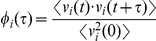
, for each direction 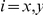 and for all possible values of 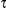, which is the lag between time points, ranging from 0 to 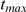 for a given trajectory. Velocity autocorrelations 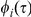 decreased exponentially, and a characteristic time constant 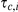 was calculated for each cell and direction as the smallest value of 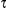 which corresponded to a value of 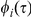 less than or equal to 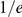.

For each step taken by a single SC, the signs of 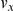 and 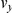 were compared to those from the previous step, and the proportion of steps in which the sign of the velocity was changed was calculated as a turning rate.

During tracking, visible interactions between SC features and substrate features were recorded and categorized as “no interaction”, “soma interaction”, and “extension interaction”. Distributions of interaction times for each microgrooved condition were determined by recording the lengths of time each SC spent in continuous contact with a substrate feature. In addition, SC movement was binned by type of visible interaction, in order to evaluate the effect that the interaction with topographical features had on alignment.

During tracking, the number of SC extensions was recorded. The average number of extensions was calculated for each SC, as was the percent of the time that each cell spent with a given number of extensions. In addition, the velocity of each SC at a given time was calculated and correlated with the number of extensions exhibited by the SC during that time interval.

### Statistical Analysis

All statistical tests were perfomed in Matlab, and a 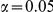 level was used to determine statistical significance. Depending on the distribution of the data being analyzed, parametric or non-parametric tests were used. Parametric tests included the two-tailed t-test and one way ANOVA. Corresponding non-parametric tests included the Mann-Whitney U (rank-sum) test and the Kruskal-Wallis one way ANOVA. The t-test and rank-sum tests were used for comparisons between two groups, and the ANOVA and Kruskal-Wallis tests were used for comparisons between three or more groups. In the latter case, a post-hoc analysis was used to test statistical significance, with the Sidak correction for multiple comparisons. Post-hoc multiple comparisons were only performed if an ANOVA or Kruskal-Wallis test was significant at the 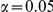 level.

## Supporting Information

Table S1
**Aligned steps (%).** Comparison of aligned movement between conditions. SC on all anisotropic conditions exhibited movement that was significantly more aligned than that found on flat, data shown graphically in Figure 2E. Following an ANOVA, post-hoc multiple comparisons with the Sidak correction were performed, 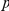-values shown.(PDF)

Table S2
**Comparison of steady state selocity between conditions.** Comparisons between conditions for steady velocity (overall, parallel, and perpendicular components), data shown graphically in Figure 3B–C. Following an ANOVA, post-hoc multiple comparisons with the Sidak correction were performed, 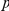-values shown.(PDF)

Table S3
**Characteristic times,** 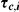 **, and comparisons with SC on flat.** Calculated values of 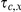 and 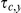, and 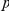-values from comparisons with 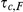, pairwise Mann-Whitney U. Data shown graphically in Figure 4B.(PDF)

Table S4
**Turning probabilities, and comparisons with SC on flat.** Calculated values of 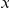 and 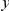 turning probabilities and 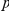-values from comparisons with the overall flat turning probability, from a Kruskal-Wallis ANOVA followed by multiple Mann-Whitney U comparisons with the Sidak correction. Data shown graphically in Figure 4C.(PDF)

Table S5
**Time spent with feature interaction (%).** Comparisons within conditions for % time spent with no contact, soma contact, or extension contact with topographical features, data shown graphically in Figure 5D. Following an ANOVA, post-hoc multiple comparisons with the Sidak correction were performed, 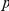-values shown.(PDF)

Table S6
**Aligned steps (%).** Comparisons within conditions for % alignment based on feature contact, for P60 and G60, data shown in Figure 5E. Following an ANOVA (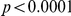), post-hoc multiple comparisons with the Sidak correction were performed, 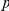-values shown.(PDF)

Table S7
**Number of extensions.** Comparisons between conditions for number of extensions, data shown in Figure 6B. Following a Kruskal-Wallis ANOVA, post-hoc multiple comparisons with the Sidak correction were performed, 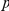-values shown.(PDF)

Figure S1
**Aligned SC movement correlated with variable contact between SC and topography.** A. Illustration of how SC contacted the feature through their soma (gray) or extension(s) (black), and when SC had no visible contact with the feature (white). B. Representative trajectories of two randomly selected cells from each condition show SC movement when SC contacted the feature through their soma (gray) or extension(s) (black), and when SC had no visible contact with the feature (white). Tick marks are spaced 50 µm apart. Dashed lines indicate the location of the edges and walls of the plateaus and grooves, respectively. C. SC on plateaus and grooves exhibited variable lengths of time of continuous contact with features. Boxplot shows lengths of time during which SC continuously contacted a feature. ‘+’ indicates mean interaction times, tops and bottoms of boxes indicate upper and lower quartiles, bands indicate medians, whiskers indicate minimum and maximum values, and dots indicate outliers. *,#: significant in Kruskal-Wallis ANOVA followed by multiple Mann-Whitney U comparisons with Sidak correction. D. Bar graph of percentage of total trajectory spent with each type of contact between SC and the features. *,#,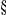: significant in comparisons within the conditions for % time spent with no contact, soma contact, or extension contact, by post-hoc multiple comparisons with the Sidak correction. E. Bar graph of percentage of aligned steps in each condition, categorized by type of contact between SC and the features. *,#: significant in comparisons within the conditions for % alignment based on feature contact, by post-hoc multiple comparisons with the Sidak correction. F. Table shows p-values for comparisons across the conditions for % alignment based on feature contact. Following an ANOVA (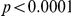), post-hoc multiple comparisons with the Sidak correction were performed. Error bars are SEM.(EPS)
