# Supplementary material for: Cellular Scale Anisotropic Topography Guides Schwann Cell Motility
Source: PLoS One. 2011 Sep 20;6(9):e24316. doi: 10.1371/journal.pone.0024316 (PMC3176770; doi:10.1371/journal.pone.0024316)
Supplement: Table S1 — Aligned steps (%). Comparison of aligned movement between conditions. SC on all anisotropic conditions exhibited movement that was significantly more aligned than that found on flat, data shown graphically in Figure 2E. Following an ANOVA, post-hoc multiple comparisons with the Sidak correction were performed, -values shown. (PDF) [file pone.0024316.s001.pdf]

Table S1. Aligned steps (%)

p-values	Flat	P30	P60	G30	G60
Flat	x	<0.0001	<0.0001	<0.0001	<0.0001
P30		x	<0.0001	1.0000	<0.0001
P60			x	<0.0001	1.0000
G30				x	<0.0001
G60					x
