# Supplementary material for: Cellular Scale Anisotropic Topography Guides Schwann Cell Motility
Source: PLoS One. 2011 Sep 20;6(9):e24316. doi: 10.1371/journal.pone.0024316 (PMC3176770; doi:10.1371/journal.pone.0024316)
Supplement: Table S2 — Comparison of steady state selocity between conditions. Comparisons between conditions for steady velocity (overall, parallel, and perpendicular components), data shown graphically in Figure 3B–C. Following an ANOVA, post-hoc multiple comparisons with the Sidak correction were performed, -values shown. (PDF) [file pone.0024316.s002.pdf]

Table S2. Comparison of steady state velocity between conditions

$\langle v_r \rangle$

p-values	Flat	P30	P60	G30	G60
Flat	x	0.0128	<0.0001	0.9838	0.9691
P30		x	0.4677	0.0074	0.0003
P60			x	<0.0001	<0.0001
G30				x	1.0000
G60					x

$\langle v_x \rangle$

p-values	Flat	P30	P60	G30	G60
Flat	x	<0.0001	0.0006	<0.0001	<0.0001
P30		x	0.0129	0.0096	0.2043
P60			x	<0.0001	<0.0001
G30				x	0.5400
G60					x

$\langle v_y \rangle$

p-values	Flat	P30	P60	G30	G60
Flat	x	<0.0001	<0.0001	<0.0001	<0.0001
P30		x	<0.0001	0.9787	<0.0001
P60			x	0.0007	0.0001
G30				x	<0.0001
G60					x
