# Supplementary material for: Cellular Scale Anisotropic Topography Guides Schwann Cell Motility
Source: PLoS One. 2011 Sep 20;6(9):e24316. doi: 10.1371/journal.pone.0024316 (PMC3176770; doi:10.1371/journal.pone.0024316)
Supplement: Table S3 — Characteristic times, , and comparisons with SC on flat. Calculated values of and , and -values from comparisons with , pairwise Mann-Whitney U. Data shown graphically in Figure 4B. (PDF) [file pone.0024316.s003.pdf]

Table S3. Characteristic times, $\tau_{c,i}$, and comparisons with SC on flat

	$\tau_{c,x}$ (min)	p-value
Flat	16.84±13.62	-
P30	39.19±41.87	<0.001
P60	22.85±26.68	0.0326
G30	26.10±24.64	0.0035
G60	19.71±15.38	0.1275

	$\tau_{c,y}$ (min)	p-value
Flat	16.84±13.62	-
P30	6.0±2.0	<0.001
P60	6.1±2.4	<0.001
G30	5.1±0.8	<0.001
G60	5.6±1.8	<0.001
