# Supplementary material for: Cellular Scale Anisotropic Topography Guides Schwann Cell Motility
Source: PLoS One. 2011 Sep 20;6(9):e24316. doi: 10.1371/journal.pone.0024316 (PMC3176770; doi:10.1371/journal.pone.0024316)
Supplement: Table S4 — Turning probabilities, and comparisons with SC on flat. Calculated values of and turning probabilities and -values from comparisons with the overall flat turning probability, from a Kruskal-Wallis ANOVA followed by multiple Mann-Whitney U comparisons with the Sidak correction. Data shown graphically in Figure 4C. (PDF) [file pone.0024316.s004.pdf]

Table S4. Turning probabilities, and comparisons with SC on flat

	x probability	p-value
Flat	0.28±0.084	-
P30	0.18±0.076	<0.001
P60	0.23±0.066	<0.001
G30	0.22±0.099	<0.001
G60	0.25±0.068	0.0190

	y probability	p-value
Flat	0.28±0.084	-
P30	0.41±0.048	<0.001
P60	0.32±0.066	<0.010
G30	0.46±0.065	<0.001
G60	0.36±0.074	<0.001
