# Supplementary material for: Cellular Scale Anisotropic Topography Guides Schwann Cell Motility
Source: PLoS One. 2011 Sep 20;6(9):e24316. doi: 10.1371/journal.pone.0024316 (PMC3176770; doi:10.1371/journal.pone.0024316)
Supplement: Table S5 — Time spent with feature interaction (%). Comparisons within conditions for % time spent with no contact, soma contact, or extension contact with topographical features, data shown graphically in Figure 5D. Following an ANOVA, post-hoc multiple comparisons with the Sidak correction were performed, -values shown. (PDF) [file pone.0024316.s005.pdf]

Table S5. Time spent with feature interaction (%)

	p-values	None	Soma	Ext.
P30	None	x	<0.0001	0.2424
	Soma		x	<0.0001
	Ext.			x
P60	None	x	0.0764	1.0000
	Soma		x	0.0531
	Ext.			x
G30	None	x	<0.0001	<0.0001
	Soma		x	0.0163
	Ext.			x
G60	None	x	<0.0001	<0.0001
	Soma		x	0.9996
	Ext.			x
