# Supplementary material for: Cellular Scale Anisotropic Topography Guides Schwann Cell Motility
Source: PLoS One. 2011 Sep 20;6(9):e24316. doi: 10.1371/journal.pone.0024316 (PMC3176770; doi:10.1371/journal.pone.0024316)
Supplement: Table S6 — Aligned steps (%). Comparisons within conditions for % alignment based on feature contact, for P60 and G60, data shown in Figure 5E. Following an ANOVA (), post-hoc multiple comparisons with the Sidak correction were performed, -values shown. (PDF) [file pone.0024316.s006.pdf]

Table S6. Aligned steps (%)

	p-values	None	Soma	Ext.
P60	None	x	<0.0001	0.2760
	Soma		x	<0.0001
	Ext.			x
G60	None	x	0.0001	<0.0001
	Soma		x	<0.0001
	Ext.			x
