# Supplementary material for: Cellular Scale Anisotropic Topography Guides Schwann Cell Motility
Source: PLoS One. 2011 Sep 20;6(9):e24316. doi: 10.1371/journal.pone.0024316 (PMC3176770; doi:10.1371/journal.pone.0024316)
Supplement: Table S7 — Number of extensions. Comparisons between conditions for number of extensions, data shown in Figure 6B. Following a Kruskal-Wallis ANOVA, post-hoc multiple comparisons with the Sidak correction were performed, -values shown. (PDF) [file pone.0024316.s007.pdf]

Table S7. Number of extensions

p-values	Flat	P30	P60	G30	G60
Flat	x	0.0453	0.9999	0.0569	0.9999
P30		x	0.0032	1.0000	0.0065
P60			x	0.0027	1.0000
G30				x	0.0057
G60					x
